# Genetic basis of the oil biosynthesis in ultra-high-oil maize grains with an oil content exceeding 20%

**DOI:** 10.3389/fpls.2023.1168216

**Published:** 2023-05-12

**Authors:** Meijie Luo, Baishan Lu, Yaxing Shi, Yanxin Zhao, Junling Liu, Chunyuan Zhang, Yuandong Wang, Hui Liu, Yamin Shi, Yanli Fan, Li Xu, Ronghuan Wang, Jiuran Zhao

**Affiliations:** Beijing Key Laboratory of Maize DNA Fingerprinting and Molecular Breeding, Maize Research Center, Beijing Academy of Agriculture and Forestry Sciences, Beijing, China

**Keywords:** ultra-high-oil maize, grain oil content, BSA-seq, BSR-seq, KASP marker, sweet maize

## Abstract

Vegetable oil is an important part of the human diet and has multiple industrial uses. The rapid increase in vegetable oil consumption has necessitated the development of viable methods for optimizing the oil content of plants. The key genes regulating the biosynthesis of maize grain oil remain mostly uncharacterized. In this study, by analyzing oil contents and performing bulked segregant RNA sequencing and mapping analyses, we determined that *su1* and *sh2-R* mediate the shrinkage of ultra-high-oil maize grains and contribute to the increase in the grain oil content. Functional kompetitive allele-specific PCR (KASP) markers developed for *su1 and sh2-R* detected *su1su1Sh2Sh2*, *Su1Su1sh2sh2*, and *su1su1sh2sh2* mutants among 183 sweet maize inbred lines. An RNA sequencing (RNA-seq) analysis indicated that genes differentially expressed between two conventional sweet maize lines and two ultra-high-oil maize lines were significantly associated with linoleic acid metabolism, cyanoamino acid metabolism, glutathione metabolism, alanine, aspartate, and glutamate metabolism, and nitrogen metabolism. A bulk segregant analysis and sequencing (BSA-seq) analysis identified another 88 genomic intervals related to grain oil content, 16 of which overlapped previously reported maize grain oil-related QTLs. The combined analysis of BSA-seq and RNA-seq data enabled the identification of candidate genes. The KASP markers for *GRMZM2G176998* (putative WD40-like beta propeller repeat family protein), *GRMZM2G021339* (homeobox-transcription factor 115), and *GRMZM2G167438* (3-ketoacyl-CoA synthase) were significantly related to maize grain oil content. Another candidate gene, *GRMZM2G099802* (GDSL-like lipase/acylhydrolase), catalyzes the final step of the triacylglycerol synthesis pathway and was expressed at significantly higher levels in the two ultra-high-oil maize lines than in the two conventional sweet maize lines. These novel findings will help clarify the genetic basis of the increased oil production in ultra-high-oil maize lines with grain oil contents exceeding 20%. The KASP markers developed in this study may be useful for breeding new high-oil sweet maize varieties.

## Introduction

Vegetable oil contains diverse fatty acids and is a renewable resource. It is an important component of the human diet and a source of industrial fuel (Zafar et al., 2019; Kim, 2020; Miklaszewska et al., 2021). By 2050, the global demand for vegetable oil is expected to be twice the current production level (Zafar et al., 2019). To meet the increasing demand for vegetable oil, methods for increasing the oil content of oilseed crops must be developed. There has been substantial biotechnology-based research on maximizing the total oil content of *Arabidopsis* and *Brassica napus* seeds. For example, the yield-related gene *fatty acid exporter 1-1* (*BnaFAX1-1A*) was identified in a genome-wide association study. Its overexpression reportedly significantly increases the *B. napus* seed oil content and yield (Xiao et al., 2021). The overexpression of the yeast gene encoding a cytoplasmic glycerol-3-phosphate dehydrogenase (*gpd1*) in *B. napus* can lead to a 3- to 4-fold increase in the glycerol-3-phosphate level and a 40% increase in the seed lipid content (Vigeolas et al., 2007). The overexpression of *miR319a* alters the seed oil composition of transgenic *Arabidopsis*, resulting in a 5.2% increase in the linoleic acid content and a 24.7% decrease in the oleic acid content (Wang et al., 2020).

The commercialization of maize grain oil has altered the trends in edible oil consumption. Its output and consumption have increased annually. Compared with other vegetable oils, maize grain oil has a higher nutritional value. More specifically, its campesterol and total phytosterol contents are high, which may help to decrease cholesterol levels and the risk of cardiovascular diseases in humans (Yang et al., 2018). The oil in maize grains, which is located mainly in the embryo, is typically a mixture of palmitic (16:0), stearic (18:0), oleic (18:1), linoleic (18:2), and linolenic (18:3) acids (Fang et al., 2021), with relatively little saturated fatty acids (11% 16:0 and 2% 18:0) and polyunsaturated fatty acids (0.7% 18:3) regardless of the maize genotype (Alrefai et al., 1995). Unsaturated fatty acids (18:1 and 18:2) account for a large proportion of the maize grain oil, with 18:1 level ranging from 14% to 64% among maize genotypes and 18:2 levels ranging from 19% to 71% (Alrefai et al., 1995). The oil content of maize grains is normally 3%–5%, but high-oil maize plants produce grains with oil contents exceeding 6%. Since 1986, high-oil maize lines have been developed and selected via conventional breeding methods. The first high-oil stocks (i.e., Illinois high-oil) had an oil content of 20% and were obtained through 100 generations of selection (Laurie et al., 2004; Hill, 2005). Subsequently, Beijing high-oil maize stocks with an oil content of 15.55% were obtained through 18 generations of directional selection (Yang et al., 2010). Clarifying the molecular mechanism underlying the high oil contents of maize grains will help to accelerate the breeding of new high-oil maize varieties.

Lipid metabolism in plants has been studied for more than 40 years (Kim, 2020). Plant fatty acids are synthesized from acetyl coenzyme A (CoA) precursors in plastids through reactions catalyzed by key enzymes, including acetyl-CoA carboxylase, malonyl CoA-acyl carrier protein transacylase, and the fatty acid synthase complex. Unsaturated fatty acids are derived from reactions mediated by stearoyl acyl carrier protein desaturase (SAD). Both saturated and unsaturated fatty acids are released from acyl carrier proteins through the action of acyl-ACP thioesterase and then exported to the cytoplasm. Fatty acids are transported to the endoplasmic reticulum, wherein they are converted to triacylglycerols (TAGs) in reactions catalyzed by several enzymes, including glycerol-phosphate acyltransferase (GPAT), lysophosphatidic acid acyltransferase, and acyl-CoA:diacylglycerol acyltransferase (DGAT). Finally, mature TAGs are transported and stored in subcellular structures called oil bodies (Liu N. et al., 2022). Transcription factors are also a crucial part of the network regulating plant oil accumulation. Studies have shown that *Leafy cotyledon1/2* (*LEC1/2*), *Fusca3* (*FUS3*), *Wrinkled1* (*WRI1*), and *Abscisic acid insensitive3* (*ABI3*) encode transcription factors that regulate the seed oil content in multiple species (e.g., *B. napus*, *Arabidopsis*, and soybean) (Li et al., 2013; Liu N. et al., 2022).

The biological processes involving hundreds of genes that lead to the synthesis of *Arabidopsis* seed oil have been thoroughly investigated (Miklaszewska et al., 2021). In contrast, only a few genes regulating the oil content and fatty acid composition of maize grains have been cloned. Specifically, *DGAT1-2* was identified via genetic mapping; the ectopic expression of the high-oil *DGAT1-2* allele in maize can increase the grain oil content and oleic acid content by 41% and 107%, respectively (Zheng et al., 2008; Chai et al., 2012). A selection pattern analysis revealed that *DGAT1-2* in maize originated in teosinte, was lost during conventional maize breeding, and was re-selected during the breeding of high-oil maize lines (Zheng et al., 2008; Fang et al., 2020). The overexpression of *ZmWRI1* or *ZmLEC1* driven by the embryo-specific promoter in maize significantly increases the maize grain oil content (Shen et al., 2010). A gene association analysis indicated that natural variations in *SAD* are related to the C18:0/C18:1 ratio in maize grains (Han et al., 2017). Using 180 individuals from the KUI3/SC55 recombinant inbred line population and high-density SNP markers, researchers identified 62 oil component-related QTLs that can explain 6.70%–31.02% of the phenotypic variation (Fang et al., 2021). In another study, 1.03 million SNPs and 368 maize inbred lines were used for a genome-wide association study that resulted in the identification of 74 QTLs significantly related to grain oil concentration or fatty acid composition, with one-third of the associated candidate genes encoding enzymes related to the oil metabolic pathway (Li et al., 2013). Additionally, the maize grain oil content is significantly affected by the embryo-to-endosperm ratio, which is influenced by *ZmGE2* (Zhang et al., 2012). Starch synthesis mutant genes, such as *su1* and *sh2*, decrease the maize endosperm size by inhibiting starch synthesis (Flora and Wiley, 1972; Dinges et al., 2001; Hu et al., 2021). However, the key enzymes and transcription factors that regulate maize grain oil biosynthesis remain mostly unknown.

Through traditional breeding, researchers have developed ultra-high-oil maize plants with a grain oil content greater than 25% (i.e., 5% higher than the grain oil content of Illinois high-oil maize). Dissecting the molecular mechanism underlying the oil production in ultra-high-oil maize will provide crucial insights into the genetic basis of maize grain oil biosynthesis, with potential implications for the breeding and genetic improvement of high-oil maize.

## Materials and methods

### Plant materials and phenotypic analysis

The 184 maize inbred lines used in this study, including 183 sweet maize inbred lines (**Supplementary Table 1**) and one common maize inbred line (B73), were obtained from the Maize Research Center of the Beijing Academy of Agriculture and Forestry Sciences (BAAFS). Among the 183 inbred lines of sweet maize, HuajianF and Huada165 are ultra-high-oil lines, but the grain oil content of HuajianF is higher than that of Huada165. Both lines were used to analyze the molecular mechanism underlying the formation of ultra-high-oil maize. The sweet maize inbred line T9 carries the *su1su1* mutation, whereas SH251 carries the *sh2sh2* mutation. These two lines were used for comparisons and analyses of the phenotypic characteristics of ultra-high-oil maize grains. The genome of common maize B73 has been sequenced and has been used as a reference genome for maize. A genetic segregation population was constructed using B73 and ultra-high-oil maize HuajianF for the genetic mapping of high oil content traits in grains. The sand culture method was used for the germination test conducted according to the National Standards of the People’s Republic of China (GB/T 3543.1-1995). The maize grain oil content (water content less than 15%) was determined using a nuclear magnetic resonance oil content analyzer (Niumag Analytical Instrument Co., Ltd., Suzhou, China). The maize grain fatty acid content was measured according to the National Standards of the People’s Republic of China (GB5009.168-2016). Briefly, the lipids in maize grains were obtained via hydrolysis followed by extraction using an ether solution, after which they were saponified and methyl esterified under alkaline conditions and analyzed using the Nexis GC-2030 gas chromatography system (Shimazdu, Kyoto, Japan). The maize grain sugar content was analyzed using the Total Sugar Content Determination kit (Solarbio Science and Technology Co., Ltd., Beijing, China). The Dionex ICS-5000+ ion chromatography system (Zhu et al., 2019) (Thermo Fisher Scientific, Waltham, MA, USA) equipped with the Dionex CarboPac PA-10 column (4 × 250 mm; Dionex) was used to analyze the water-soluble sugar components in maize grains. The mobile phase consisted of eluent A (ultrapure water) and eluent B (250 mmol/L NaOH), and the injection volume was 25 µl. The maize grains used for these analyses were harvested after maturity and then dried to a water content below 15%. The analyses were performed using at least three biological replicates.

### Bulked segregant RNA sequencing

An F_2_ segregating population was derived from the B73 × HuajianF hybridization. A nuclear magnetic resonance oil content analyzer (Niumag Analytical Instrument Co., Ltd., Suzhou, China) was used to determine the oil content of maize grains. The oil content of the shrunken (non-transparent) F_2_ grains was 7.66% higher than that of the round F_2_ grains. In addition, the oil content of the transparent F_2_ grains was 4.20% higher than that of the round F_2_ grains (the transparent grains were also shrunken, but they were called transparent to distinguish them from the non-transparent shrunken grains). Thirty round and 30 shrunken F_2_ grains were used for sprouting to map the gene mediating the shrinkage phenotype. Additionally, 30 non-transparent and 30 transparent F_2_ grains were used for sprouting to map the gene controlling the transparency phenotype. Total RNA was extracted from the four pooled leaf samples using TRIzol as previously described (Luo et al., 2022) for the subsequent sequencing on the Illumina NovaSeq 6000 system (Illumina). The sequencing data were filtered using Trimmomatic software to eliminate reads with a Phred quality score less than 15 (Bolger et al., 2014). The clean reads were aligned with the B73 reference genome (RefGen_v3) using the Genomic Short-Read Nucleotide Alignment Program (Wu et al., 2016). The Genome Analysis Toolkit (GATK) was used for detecting SNPs in uniquely aligned reads (Mckenna et al., 2010). The probability of a linkage between a SNP and the causal gene was calculated according to the Bayesian method (Liu et al., 2012). The associated SNPs were identified on the basis of a linkage probability value greater than 0.05.

### Sequence analysis

Total genomic DNA of maize lines HuajianF, Huada165, and B73 was extracted from leaves using the Plant Genomic DNA Extraction Kit (Tiangen, Beijing, China). Total RNA of maize lines HuajianF, Huada165, and B73 was extracted from leaves using TRIzol (Luo et al., 2022) and then reverse transcribed to synthesize cDNA using the PrimeScript™ II 1st Strand cDNA Synthesis Kit (Takara, Shiga, Japan). The PCR amplification was completed using Super-Fidelity DNA polymerase (Vazyme Biotech, China) and gene-specific primers (**Supplementary Table 2**). The amplified products were sequenced using the ABI3730 sequencer (Tianyi Huiyuan Biotech, Beijing, China). The sequences were aligned using the MegAlign software (http://www.freedownload64.com/s/megalign).

### Bulk segregant analysis sequencing

The Plant Genomic DNA Extraction Kit (Tiangen) was used to extract total genomic DNA from 903 plants in the F_2_ population derived from the HuajianF × Huada165 hybridization. In addition, the oil content of the F_2:3_ grains produced in F_2_ maize ears by selfing was determined using a nuclear magnetic resonance oil content analyzer (Niumag Analytical Instrument Co., Ltd.). The analysis was performed using four biological replicates. Two DNA pools were constructed using leaf genomic DNA from 46 F_2_ plants with the highest grain oil contents and 46 F_2_ plants with the lowest grain oil contents. These two DNA pools and the DNA of the two parents were sequenced using the NovaSeq 6000 system (Illumina). The clean reads were aligned with the maize B73 reference genome (RefGen_v4) using the Burrows–Wheeler aligner (Li and Durbin, 2009). Variants were called using the Unified Genotyper of GATK, and all mutations were annotated using ANNOVAR (Wang et al., 2010). Moreover, SNP-index and Euclidean distance (ED) methods were used for association analysis. Genes were annotated according to a BLAST search of multiple databases (e.g., the NR, Swiss-Prot, GO, KEGG, and KOG databases).

### RNA sequencing

Total RNA was extracted from the grains of maize inbred lines HuajianF, Huada165, T9, and SH251 using TRIzol (Luo et al., 2022) at 23 days after self-pollination. Three biological replicates were prepared, with the grains from five plants combined into one biological replicate. The RNA was sequenced using the NovaSeq 6000 system (Illumina). The trimmed clean reads were aligned with the B73 maize reference genome (RefGen_v3) using HISAT2 software (Kim et al., 2015). Differentially expressed genes (DEGs) were identified using DEseq2 (Love et al., 2014). Significant DEGs were screened using the following criteria: expression-level fold-change ≥2 and FDR <0.01. The KOBAS software was used to identify enriched Kyoto Encyclopedia of Genes and Genomes (KEGG) pathways among the DEGs (Chen et al., 2011).

### Kompetitive allele-specific PCR assay

The KASP primers (**Supplementary Table 2**) were designed for SNPs or insertion/deletion (InDel) sites in oil content-regulating genes or candidate genes. The KASP assay was conducted using a high-throughput genotyping platform (LGC, Middlesex, UK) (Tang et al., 2021).

### Statistical analysis

Unpaired two-tailed Student’s *t-*tests were performed using GraphPad Prism 9 (http://www.graphpad.com/) to evaluate the significance of any differences. Data were recorded as the mean ± SEM.

## Results

### Ultra-high-oil maize grains have high oil and sugar contents

**Figure 1:**
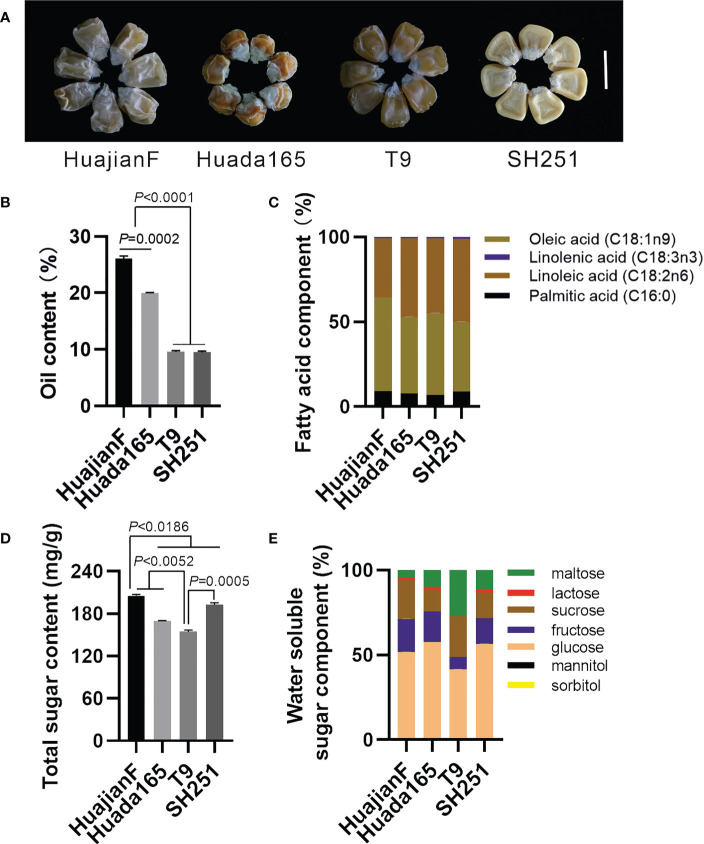
Grain oil and sugar contents in the ultra-high-oil maize lines HuajianF and Huada165 and the conventional sweet maize lines T9 and SH251. The water content of the tested grains was lower than 15%. **(A)** HuajianF, Huada165, T9, and SH251 grains. Scale bar = 1 cm. **(B)** Oil content. **(C)** Fatty acid composition. **(D)** Total sugar content. **(E)** Water-soluble sugar composition. Three biological replicates were analyzed. Unpaired two-tailed Student’s *t-*tests were used to determine the significance of any differences. Data are presented as the mean ± SEM.

The ultra-high-oil maize inbred lines HuajianF and Huada165 were obtained by conventional breeding. Conventional sweet maize inbred lines T9 and SH251, which are commonly used for maize breeding, carry the recessive *su1* and *sh2* genes, respectively. Both HuajianF and Huada165 had grain germination rates greater than 98% (**Supplementary Figure 1**). The harvested grains of HuajianF, Huada165, T9, and SH251 exhibited shrinkage characteristics (**Figure 1A**), and their oil contents were 26.08 ± 0.47%, 19.94 ± 0.13%, 9.56 ± 0.22%, and 9.46 ± 0.21%, respectively. Thus, the oil contents of the HuajianF and Huada165 grains were more than twice those of the T9 and SH251 grains. The oil content of the HuajianF grains was 6.14% higher than that of the Huada165 grains (**Figure 1B**). The analysis of the fatty acid composition revealed that oleic acid (18:1) and linoleic acid (18:2) accounted for nearly 90% of the oil components in the four maize inbred lines, whereas palmitic acid (16:0) and linolenic acid (18:3) accounted for 7.02%–9.12% and 0.80%–1.25% of the oil components, respectively (**Figure 1C**). The total sugar content was highest in HuajianF (205.2 ± 1.94 mg/g) and lowest in T9 (154.3 ± 2.63 mg/g) (**Figure 1D**). The main water-soluble sugars in the four maize lines were glucose, fructose, sucrose, and maltose. The proportion of maltose was higher in T9 (27.18%) than in the other three maize lines (4.66%–11.44%). Furthermore, lactose was undetectable in T9 (**Figure 1E**).

### *sh2* contributes to the oil content of ultra-high-oil maize grains

**Figure 2:**
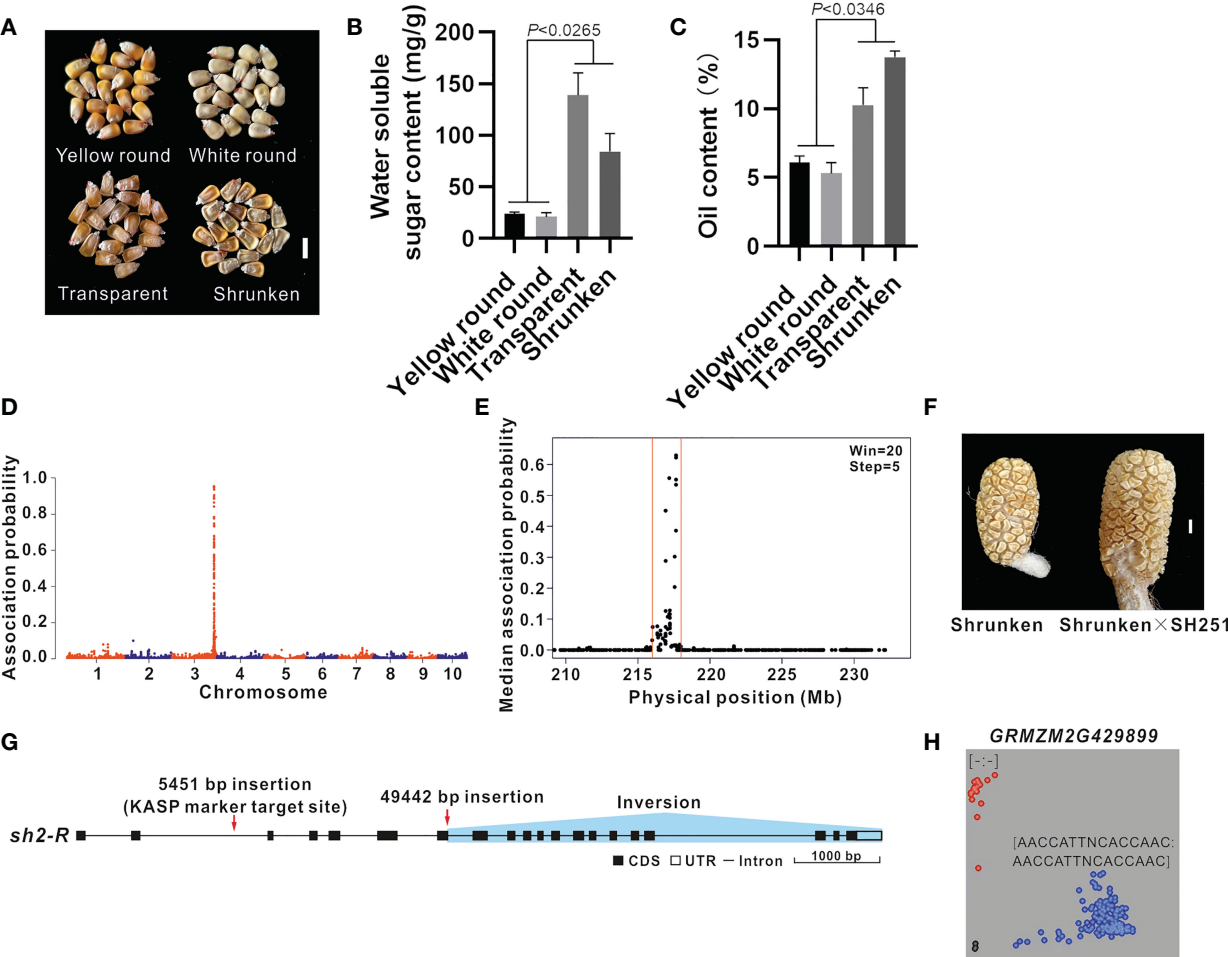
BSR-seq mapping and functional molecular marker development for the gene related to the grain shrinkage phenotype. **(A)** Phenotype of the F_2_ grains (B73 × HuajianF). There were four grain types: yellow round, white round, transparent, and shrunken. Scale bar = 1 cm. **(B, C)** Water-soluble sugar content **(B)** and oil content **(C)** among the different types of F_2_ grains (B73 × HuajianF). Three biological replicates were analyzed. **(D)** BSR-seq mapping of the gene regulating the grain shrinkage phenotype to chromosome 3. **(E)** The gene regulating the grain shrinkage phenotype was mapped to the 216–218 Mb region on chromosome 3 (B73 RefGen_v3). **(F)** Allelism test of *sh2* mutants. Shrunken F_2_ grains (B73 × HuajianF) (left) and the grains of the hybrid derived from the cross with SH251 (right). Scale bar = 1 cm. **(G)** *sh2* sequence variations in HuajianF. **(H)** Genotyping results for 183 sweet maize inbred lines using *the sh2* functional KASP marker, which was developed according to the 5,451-bp insertion in *sh2*. Unpaired two-tailed Student’s *t-*tests were used to determine the significance of any differences. Data are presented as the mean ± SEM.

Maize inbred line B73 (4.05 ± 0.16% grain oil content) was crossed with the ultra-high-oil maize line HuajianF. The resulting F_2_ grains were classified as follows: yellow round, white round, transparent, and shrunken (**Figures 2A, B**), with oil contents of 6.07 ± 0.49%, 5.30 ± 0.78%, 10.27 ± 1.24%, and 13.73 ± 0.44%, respectively (**Figure 2C**). Accordingly, the grain oil contents were significantly higher in the transparent and shrunken grains than in the round grains, reflecting the relationship between the grain oil content and grain transparency and shrinkage.

In the current study, BSR-seq mapping was performed to identify the genes regulating the grain oil content based on the grain shrinkage phenotype (7.66% increase in the grain oil content). Two RNA pools were prepared from 30 shrunken F_2_ grains and 30 round F_2_ grains. A total of 202 million reads were generated by RNA-seq. After the reads were trimmed and aligned and SNPs were called (Luo et al., 2022), 229,417 SNPs remained. Only the SNPs associated with 11 reads (at least three reads for each allele in the round F_2_ grain pool and at least five reads for two alleles in the shrunken F_2_ grain pool) were used for identifying causal loci. The empirical Bayesian method was used to analyze the probability of an association between the high-confidence SNPs and the causal gene. A total of 211 SNPs had an association probability greater than 0.05, of which 97% were on chromosome 3 (**Figure 2D**). These SNPs were scanned using the sliding window method (window, 20 SNPs; step, five SNPs). The candidate gene was mapped to the 216–218 Mb region on chromosome 3 of the B73 reference genome (B73 RefGen_v3) (**Figure 2E**). On the basis of the functional annotation of the 65 genes in this region, *GRMZM2G429899* was identified as the *Shrunken 2* (*Sh2*) gene. A loss-of-function mutation in this gene reportedly leads to the production of shrunken maize grains (Kramer et al., 2015). The F_2_ plants with shrunken grains were crossed with maize line SH251 (carrying the *sh2sh2* homozygous mutation). The resulting hybrids produced shrunken grains (**Figure 2F**). The results of the *sh2* allelism test for the F_2_ grains (B73 × HuajianF) indicated that the shrunken grain phenotype was controlled by *sh2*, which was derived from HuajianF.

There were no PCR amplification products for the second intron and exons 6–8 of *sh2* in HuajianF, indicative of the sequence variations in these regions (**Supplementary Figure 2**). The genome of the sweet maize inbred line Ia453 carrying the mutated *sh2-R* gene has been sequenced (Hu et al., 2021). Its *sh2-R* allele contains a 5,451-bp insertion mutation in the second intron, a 49,442-bp insertion mutation after the 121st nucleotide of the seventh exon, and an inversion mutation from the 121st nucleotide of the seventh exon to the 20th exon. These results indicate that the *sh2* allele in HuajianF is *sh2-R* (**Figure 2G**). Based on the 5,451-bp insertion mutation, a KASP marker for *sh2-R* was developed to genotype 183 sweet maize inbred lines used in modern breeding programs (**Supplementary Table 1**). The genotyping results suggested that 169 of the examined sweet maize inbred lines carry the *sh2-R* homozygous mutation (**Figure 2H**), which was consistent with our expectations. Hence, the KASP marker may be used to accurately and quickly identify maize lines carrying *sh2-R*.

### *su1* contributes to the oil content of ultra-high-oil maize grains

**Figure 3:**
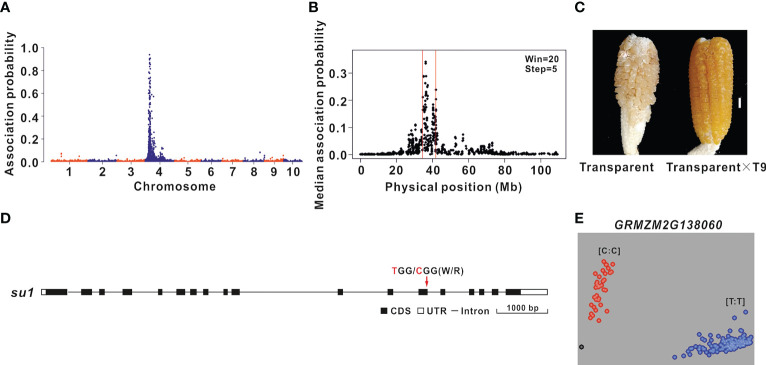
BSR-seq mapping and functional molecular marker development for the gene related to the grain transparency phenotype. **(A)** BSR-seq mapping of the gene regulating the F_2_ grain (B73 × HuajianF) transparency phenotype to chromosome 4. **(B)** The gene regulating the F_2_ grain (B73 × HuajianF) transparency phenotype was mapped to the 35–42 Mb region on chromosome 4 (B73 RefGen_v3). **(C)** Allelism test of *su1* mutants. Transparent F_2_ grains (B73 × HuajianF) (left) and the grains of the hybrid derived from the cross with T9 (right). Scale bar = 1 cm. **(D)** *su1* sequence variations in HuajianF. **(E)** Genotyping results for 183 sweet maize inbred lines using *the su1* functional KASP marker, which was developed according to the SNP (T-to-C) in *su1*.

The oil content of transparent F_2_ grains was 4.20% higher than that of the round F_2_ grains. Therefore, BSR-seq mapping was performed to identify the genes regulating the grain oil content of the ultra-high-oil maize line HuajianF according to the transparency of F_2_ grains (B73 × HuajianF). Two RNA pools were prepared from 30 transparent F_2_ grains and 30 non-transparent F_2_ grains. A total of 164 million reads were generated by RNA-seq. A total of 500 SNPs with an association probability exceeding 0.05 were detected, of which 99% were on chromosome 4 (**Figure 3A**). Using the sliding window method, the candidate gene was mapped to the 35–42 Mb region on chromosome 4 (B73 RefGen_v3) (**Figure 3B**), which included 147 genes. The functional annotation of these genes identified *GRMZM2G138060* as the *Sugary 1* (*Su1*) gene, which is critical for maize grain transparency (Dinges et al., 2001). The F_2_ plants with transparent grains were crossed with maize line T9 (carrying the *su1su1* homozygous mutation). The grains of the generated hybrids were transparent (**Figure 3C**). The *su1* allelism test results suggested that the transparency of the F_2_ grains (B73 × HuajianF) was due to *su1*, which was derived from HuajianF. The analysis of the *su1* sequence in HuajianF revealed a T-to-C (Trp-to-Arg) mutation at the 145th nucleotide of the 13th exon (**Figure 3D**). A previous study demonstrated that this SNP results in a non-functional *Su1* (Dinges et al., 2001). Accordingly, on the basis of this mutation, a KASP marker for *su1* was developed and used for the genotyping of 183 sweet maize inbred lines used in modern breeding programs (**Supplementary Table 1**). The genotyping results suggested that 34 of the tested maize lines carried *the su1su1* mutation (**Figure 3E**). Thus, there were far fewer maize lines carrying *the su1su1* mutation than maize lines with the *sh2sh2* mutation; this difference may be related to market demand for *sh2sh2* sweet maize. The KASP marker for *su1* can accurately and rapidly detect the *su1* mutation in maize lines.

### Identification of QTLs related to the grain oil content of ultra-high-oil maize via BSA-seq

**Figure 4:**
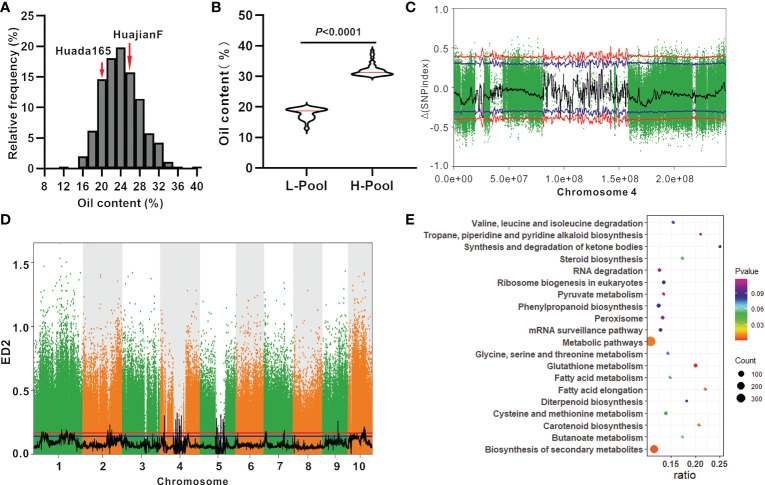
BSA-seq mapping of the oil content-regulating genes using the F_2_ population (HuajianF × Huada165). **(A)** Frequency distribution of the oil content in F_2:3_ grains (HuajianF × Huada165). **(B)** Average oil content difference between 46 F_2_ plants (HuajianF × Huada165) with the highest grain oil contents and 46 F_2_ plants (HuajianF × Huada165) with the lowest grain oil contents. Unpaired two-tailed Student’s *t-*tests were used to determine the significance of any differences. **(C)** Results of the BSA-seq mapping of the grain oil content-regulating gene based on the SNP-index association algorithm. **(D)** Results of the BSA-seq mapping of the grain oil content-regulating gene based on the Euclidean distance (ED) algorithm. **(E)** The top 20 enriched pathways among the genes in the genomic intervals mapped via BSA-seq and the ED algorithm.

The grain oil content of the ultra-high-oil maize line HuajianF was more than twice that of the sweet maize lines T9 and SH251, indicating that there are other genes encoding regulators of grain oil contents besides *su1* and *sh2*. KASP genotyping (**Supplementary Table 1**) and PCR sequencing results suggested that HuajianF and Huada165 both carry the *su1* and *sh2-R* mutations. An F_2_ segregating population comprising 903 individuals was constructed using HuajianF and Huada165 to map the oil content-regulating genes. The grain oil content of the F_2_ population ranged from 12.83% to 38.42%, with an average of 24.12% (**Figure 4A**). Genomic DNA was extracted from 46 F_2_ plants with extremely high grain oil contents (30.03%–38.42%), 46 F_2_ plants with extremely low grain oil contents (12.83%–19.90%) (**Figure 4B**), and the two parents for the BSA-seq analysis. A total of 274 Gb of high-quality, clean data were obtained. The sequencing depths of the parents and extreme-phenotype pools were 20× and 40×, respectively. The high-quality reads were aligned with the B73 RefGen_v4 sequence. A total of 10 million high-quality SNPs and 1.1 million high-quality InDels were obtained. According to the SNP-index association algorithm, four genomic regions (25.26–27.37 Mb, 96.11–97.49 Mb, 104.69–106.15 Mb, and 130.20–132.05 Mb) on chromosome 4 exceeded the threshold (95% confidence interval) (**Figure 4C**). When the ED algorithm was used, 88 genomic regions exceeded the threshold (95% confidence interval); these genomic regions were distributed on 10 chromosomes and included the four genomic regions detected using the SNP-index association algorithm (**Figure 4D**; **Supplementary Table 3**). An examination of the B73 RefGen_v4 sequence indicated that the 88 genomic regions contained 3,976 genes. The significantly enriched pathways among these genes included glutathione metabolism, biosynthesis of secondary metabolites, fatty acid elongation, carotenoid biosynthesis, and metabolic pathways (**Figure 4E**).

### Differentially expressed genes and enriched metabolic pathways in ultra-high-oil maize

**Figure 5:**
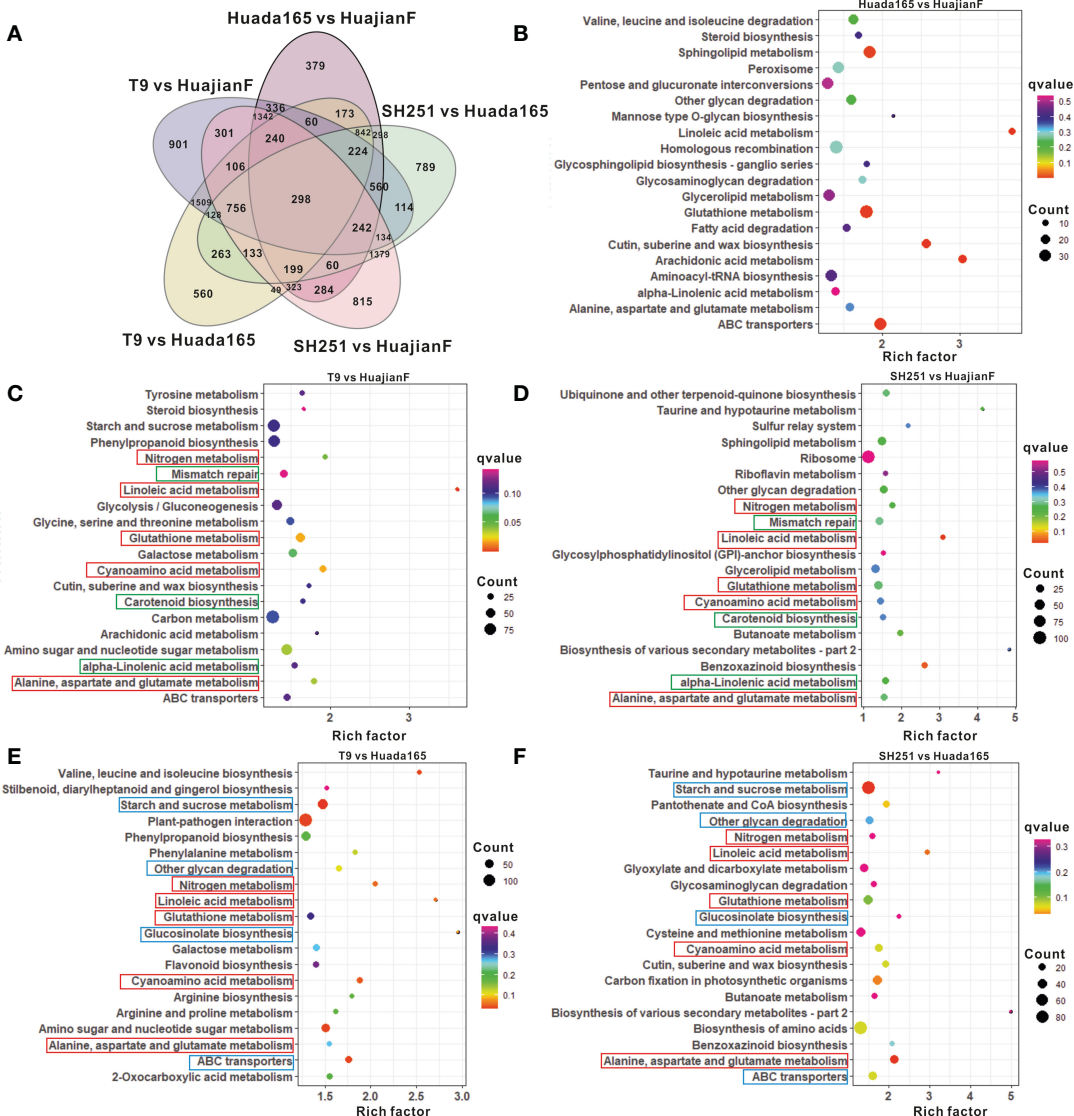
Differentially expressed genes (DEGs) and the top 20 enriched KEGG pathways. **(A)** Number of DEGs in HuajianF and Huada165. **(B–F)** Top 20 enriched KEGG pathways among the DEGs between Huada165 and HuajianF **(B)**, between T9 and HuajianF **(C)**, between SH251 and HuajianF **(D)**, between T9 and Huada165 **(E)**, and between SH251 and Huada165 **(F)**.

The grains of two ultra-high-oil maize lines (HuajianF and Huada165) and two conventional sweet maize lines (T9 and SH251) were collected 23 days after self-pollination for RNA-seq analysis. The comparison with Huada165 detected 4,906 DEGs in HuajianF (**Figure 5A**; **Supplementary Table 4**). The most enriched pathways among these DEGs were linoleic acid metabolism, glutathione metabolism, alanine, aspartate, and glutamate metabolism, and glycerolipid metabolism (**Figure 5B**). These DEGs included 2,600 significantly upregulated DEGs, of which 17 and 7 were associated with glycerolipid metabolism and fatty acid elongation, respectively.

**Figure 6:**
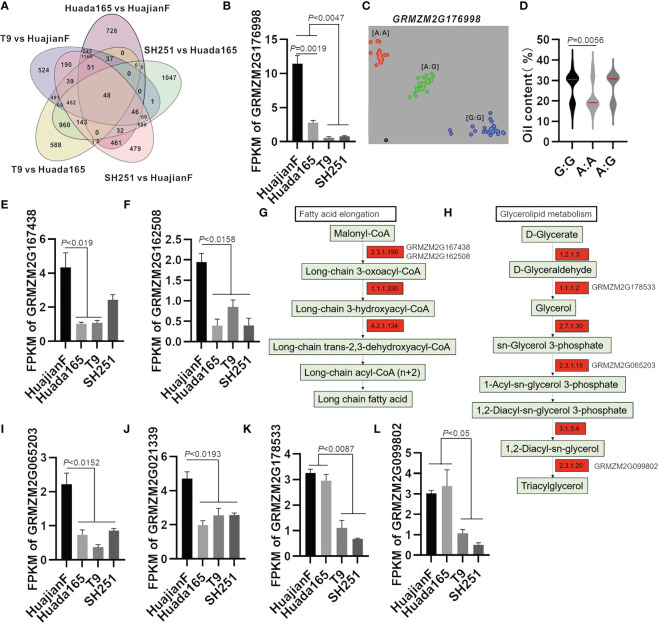
Candidate genes regulating the maize grain oil content. **(A)** Number of up-regulated DEGs in HuajianF and Huada165. **(B)** Differential expression of *GRMZM2G176998* in HuajianF, Huada165, T9, and SH251 in terms of the fragments per kilobase million (FPKM) value. **(C)** KASP marker genotyping results for *GRMZM2G176998 in* 48 F_2_ plants (HuajianF × Huada165) with an extremely low oil content and 54 F_2_ plants (HuajianF × Huada165) with an extremely high oil content. **(D)** Association between the genotyping results and the grain oil contents. **(E, F)** Differential expression of candidate genes in HuajianF, Huada165, T9, and SH251 in terms of the FPKM value: **(E)** *GRMZM2G167438* and **(F)** *GRMZM2G162508*. **(G, H)** Fatty acid elongation and glycerolipid metabolism pathway genes more highly expressed in HuajianF than in Huada165 are indicated in red. **(I–L)** Differential expression of candidate genes in HuajianF, Huada165, T9, and SH251 in terms of the FPKM value: **(I)** *GRMZM2G065203*, **(J)** *GRMZM2G021339*, **(K)** *GRMZM2G178533*, and **(L)** *GRMZM2G099802*. Unpaired two-tailed Student’s *t-*tests were used to determine the significance of any differences. Data are presented as the mean ± SEM.

The comparisons with conventional sweet maize lines T9 and SH251 revealed DEGs in the ultra-high-oil maize line HuajianF that were significantly associated with linoleic acid metabolism, cyanoamino acid metabolism, glutathione metabolism, alanine, aspartate, and glutamate metabolism, nitrogen metabolism, carotenoid biosynthesis, alpha-linolenic acid metabolism, and mismatch repair (**Figures 5C, D**). In contrast, the significantly enriched pathways among the DEGs in the ultra-high-oil maize line Huada165 revealed by the comparisons with conventional sweet maize lines were starch and sucrose metabolism, ABC transporters, cyanoamino acid metabolism, nitrogen metabolism, linoleic acid metabolism, glucosinolate biosynthesis, other glycan degradation, alanine, aspartate, and glutamate metabolism, and glutathione metabolism (**Figures 5E, F**). Accordingly, the significantly enriched metabolic pathways common to the DEGs in HuajianF and Huada165 were linoleic acid metabolism, cyanoamino acid metabolism, glutathione metabolism, alanine, aspartate, and glutamate metabolism, and nitrogen metabolism (**Figures 5C, F**). Additionally, 1,054 genes were detected as DEGs in both HuajianF and Huada165 (**Figure 5A**), of which 500 and 391 were significantly upregulated (**Figure 6A**) and downregulated, respectively. The significantly enriched pathways among the 500 upregulated DEGs were phenylpropanoid biosynthesis, flavonoid biosynthesis, stilbenoid, diarylheptanoid and gingerol biosynthesis, linoleic acid metabolism, photosynthesis, ubiquinone and other terpenoid-quinone biosynthesis, and oxidative phosphorylation. Of the 500 up-regulated DEGs, the expression levels of 48 were significantly higher in HuajianF than in Huada165 (**Figure 6A**). Thesignificantly enriched pathways associated with these 48 upregulated DEGs were alpha-linoleic acid metabolism, linoleic acid metabolism, glycosylphosphatidylinositol-anchored biosynthesis, nitrogen metabolism, and spliceosome.

### Other candidate genes affecting the oil content of ultra-high-oil maize grains

The annotated gene functions, enriched metabolic pathways, and gene expression changes were combined to screen the genomic intervals mapped via BSA-seq for the key candidate genes regulating the oil content of ultra-high-oil maize grains.

The expression levels of three genes (*Zea_mays_newGene_8111*, *GRMZM2G176998*, *and GRMZM5G830269*) at the mapped genomic intervals were significantly higher in HuajianF than in Huada165. Moreover, the expression levels of these genes were significantly higher in HuajianF and Huada165 than in T9 and SH251 (**Figures 6A, B**). Gene functional annotations indicated that *Zea_mays_newGene_8111* encodes glucose-1-phosphate adenylyltransferase large subunit 2, which is involved in starch and sugar metabolism, whereas *GRMZM2G176998* encodes a WD40-like beta propeller repeat family protein, which contributes to intracellular trafficking, secretion, and vesicular transport. The BSA-seq analysis revealed 35 SNVs (SNPs and InDels) in *GRMZM2G176998* between HuajianF and Huada165, including four InDels in the upstream region and four non-synonymous SNPs in the exon. The differences in the SNPs in *GRMZM2G176998* between the two parents were used to develop a KASP marker for genotyping 48 low-oil and 54 high-oil F_2_ plants (HuajianF × Huada165) (**Figure 6C**). The KASP marker was significantly related to the grain oil content (**Figure 6D**), indicating that *GRMZM2G176998* might encode a protein that helps regulate the grain oil content.

Both *GRMZM2G167438* and *GRMZM2G162508* encode 3-ketoacyl-CoA synthase (*KCS12* and *KCS20*, respectively), which is involved in fatty acid elongation (**Figure 6G**), whereas *GRMZM2G065203* encodes GPAT1, which helps mediate glycerolipid metabolism (**Figure 6H**). These genes, which were detected at the mapped genomic intervals, were significantly more highly expressed in HuajianF than in Huada165 (**Figures 6E–I**). They were selected as candidate genes that regulate the oil content of ultra-high-oil maize grains. The BSA-seq analysis detected 33 SNVs in *GRMZM2G167438*, including four InDels in the 3′ UTR. The KASP marker for *GRMZM2G167438* was significantly related to the maize grain oil content (**Supplementary Figures 3A, B**). The BSA-seq analysis also detected 39 SNVs in *GRMZM2G162508*, including two non-synonymous SNPs in exon 2 and 11 InDels in the upstream and downstream sequences as well as the 5′ UTR.

The *GRMZM2G021339* gene encoding homeobox transcription factor 115 was in a genomic interval (168,690,001–175,140,000) on chromosome 4. This interval overlaps with the previously identified oil-related QTL *qPALE4-2* (Fang et al., 2021). Its expression level was significantly higher in HuajianF than in Huada165 (**Figure 6J**). The KASP marker for *GRMZM2G021339* was significantly associated with grain oil content (**Supplementary Figures 3C, D**). Therefore, *GRMZM2G021339* was considered a candidate gene regulating maize grain oil content. The BSA-seq analysis detected 38 SNVs in *GRMZM2G021339*, including two InDels and seven non-synonymous SNPs in exons, as well as three InDels in the 5′ UTR and one InDel in the 3′ UTR.

The RNA-seq analysis revealed that *GRMZM2G178533* (encoding an aldo/keto reductase family member) and *GRMZM2G099802* (encoding GDSL-like lipase/acylhydrolase) were expressed at significantly higher levels in the ultra-high-oil maize lines HuajianF and Huada165 than in the conventional sweet maize lines T9 and SH251 (**Figures 6K, L**). The enzymes encoded by *GRMZM2G178533* and *GRMZM2G099802* catalyze the synthesis of TAGs in the glycerolipid metabolism pathway, indicating that these two genes are related to the increased grain oil content of ultra-high-oil maize lines.

## Discussion

Triacylglycerols, which are the major components of vegetable oil, have various important functions in diverse plant species. More specifically, they are a carbon and energy reserve, and they are also critical for cell expansion and division, stomatal opening, membrane lipid remodeling, organ formation, and pollination (Yang and Benning, 2018). Therefore, studies on optimizing TAG content in different plant tissues are underway (Kim, 2020). In the current study, a comparative transcriptome analysis was conducted to reveal the differences between ultra-high-oil and conventional sweet maize lines in terms of gene expression and the associated metabolic pathways. Combining the BSR-seq and BSA-seq analyses enabled the identification of key candidate genes regulating maize grain oil content. In addition, KASP markers were developed for the variable gene sequences. The findings of this study may provide the foundation for molecular breeding and improvement of high-oil maize lines.

Environmental conditions can influence maize grain oil content. For example, the application of N, P, and K fertilizers reportedly leads to slight increases in the oil content of maize grains (Wang et al., 2008). Previous research on the molecular mechanisms in high-oil maize plants confirmed the broad-sense heritability of maize grain oil-related traits (52%–98%) (Yang et al., 2010; Fang et al., 2021), reflecting the substantial effect of genotype on the oil content. The grain oil content is 5% higher in the ultra-high-oil maize line HuajianF than in the Illinois high-oil maize line obtained after 100 generations of selection. Morphologically, Illinois high-oil maize grains are round, which is in contrast to the shrunken HuajianF grains. The oil content (10.27%–13.73%) of the shrunken F_2_ grains (B73 × HuajianF) was significantly higher than that (5.30%–6.07%) of the round F_2_ grains (B73 × HuajianF), indicative of the relationship between grain shrinkage and increased oil contents. These observations also help to explain why the oil content was higher in HuajianF grains than in Illinois high-oil maize grains. The BSR-seq mapping and gene mutant allelism test results indicated that *su1* is responsible for the transparent and shrunken F_2_ grains (B73 × HuajianF), whereas *sh2* was associated with the non-transparent and shrunken grain phenotype. Compared with normal maize grains, sweet maize grains (containing *su1* or *sh2*) have a lower endosperm volume because of a decrease in starch synthesis (Dinges et al., 2001; Hu et al., 2021), which is one way to increase the maize grain oil content. The two functional KASP markers developed on the basis of the *su1* and *sh2* sequence variations were able to distinguish the maize lines carrying *su1su1Sh2Sh2*, *Su1Su1sh2sh2*, and *su1su1sh2sh2* among 183 modern maize inbred lines. These molecular markers may be useful for molecular marker-assisted selection-based breeding of new sweet maize varieties.

The 88 maize grain oil content-related genomic intervals that were identified by BSA-seq mapping were distributed on all maize chromosomes, indicating that ultra-high maize grain oil content is a quantitative trait controlled by multiple genes. Moreover, 18.2% (16/88) of the mapped genomic intervals overlapped QTLs identified in a previous study (Fang et al., 2021), indicative of the reliability and accuracy of our results. Candidate genes in the mapped genomic intervals were screened according to gene expression level changes, the associated metabolic pathways, and the findings of earlier studies. The WD40 repeat proteins regulate diverse biological processes (Jain and Pandey, 2018). One of the candidate genes in the mapped genomic interval (*GRMZM2G176998*) encodes a putative WD40-like beta propeller repeat family protein. Notably, its expression level was significantly higher in the two ultra-high-oil maize lines than in the two conventional sweet maize lines. The KASP markers for *GRMZM2G176998* were used to genotype 48 low-oil F_2_ plants (HuajianF × Huada165) and 54 high-oil F_2_ plants (HuajianF × Huada165). The genotyping results showed that the KASP marker for *GRMZM2G176998* was significantly related to the oil content, suggesting *GRMZM2G176998* helps regulate the maize grain oil content. Homeobox transcription factors participate in various plant developmental processes (Tsuda and Hake, 2016). The candidate gene *GRMZM2G021339*, which encodes homeobox transcription factor 115, was in the mapped genomic interval (168,690,001–175,140,000 bp) on chromosome 4. Its KASP marker was significantly related to the grain oil content of F_2_ plants (HuajianF × Huada165). Moreover, this gene was previously identified as a candidate gene regulating the C16:1 content and the C16:0/C16:1 ratio in maize grains (Fang et al., 2021). The elongation of long-chain fatty acids, which are the main components of seed TAGs, is controlled by KCS (Blacklock and Jaworski, 2006). The candidate genes *GRMZM2G167438* (*KCS12*) and *GRMZM2G162508* (*KCS20*) were among the genes in the mapped genomic interval, and the KASP marker for *GRMZM2G167438* was significantly related to the oil content of F_2_ plants (HuajianF × Huada165). A recent study confirmed that GPAT is a key rate-limiting enzyme that catalyzes the first step of the TAG biosynthesis pathway (Liu H. et al., 2022). The overexpression of *GPAT* in *Phaeodactylum tricornutum* reportedly increases the oil body volume and doubles the neutral lipid content (Niu et al., 2016). The candidate gene (*GRMZM2G065203*) encoding GPAT1 in the mapped genomic interval overlaps with the previously detected maize grain oil content-related QTL *qOIL3-2* (Fang et al., 2021). Thus, *GRMZM2G065203* might be an important candidate gene encoding an oil content regulator.

The expression of *GRMZM2G178533* (an aldo/keto reductase family member that contributes to TAG biosynthesis) was significantly higher in the two ultra-high-oil maize lines than in the two conventional sweet maize lines. An earlier transcriptome analysis showed that GDSL esterase or lipase genes are more highly expressed in high-oil sesame lines than in low-oil sesame lines (Dutta et al., 2022). Consistent with this earlier finding, *GRMZM2G099802* (encoding a GDSL-like lipase/acylhydrolase) was significantly more highly expressed in the two ultra-high-oil maize lines than in the two conventional sweet maize lines. Moreover, GDSL-like lipase/acylhydrolase catalyzes the final step of the TAG biosynthesis pathway. Hence, both *GRMZM2G178533* and *GRMZM2G099802* may encode proteins that regulate maize grain oil content. Additionally, eight genes (*GRMZM2G174766*, *AC215690.3_FG002*, *GRMZM2G073929*, *GRMZM2G394968*, *GRMZM5G848768*, *GRMZM5G860072*, *GRMZM2G090733*, and *GRMZM2G445791*) mediating unsaturated fatty acid synthesis were detected in the BSA-seq mapped genomic intervals, indicating they might be related to oil biosynthesis in ultra-high-oil maize grains.

In a previous study, the SAD-encoding *AC215690.3_FG002* gene was highly expressed in most maize embryonic developmental stages as well as in the seed and endosperm (Han et al., 2017). The RNA-seq analysis in the current study showed that *AC215690.3_FG002* was highly expressed in the grains of the four examined maize lines. The RNA-seq data also indicated that the two ultra-high-oil maize lines differed considerably from the two conventional sweet maize lines in terms of linoleic acid metabolism, cyanoamino acid metabolism, glutathione metabolism, alanine, aspartate, and glutamate metabolism, and nitrogen metabolism. Accordingly, these metabolic pathways might be related to oil metabolism. There might be a relationship between glutathione metabolism and fatty acid oxidation. Nitrogen metabolism may affect the maintenance of the C/N balance in plants (Liu et al., 2017). The highly enriched pathways associated with the 500 significantly upregulated DEGs in the two ultra-high-oil maize lines included phenylpropanoid biosynthesis, flavonoid biosynthesis, stilbenoid, diarylheptanoid and gingerol biosynthesis, linoleic acid metabolism, photosynthesis, ubiquinone and other terpenoid-quinone biosynthesis, and oxidative phosphorylation. In accordance with these findings, earlier studies on *Arabidopsis* determined that fatty acid biosynthesis and phenylpropanoid biosynthesis are closely associated (Vogt, 2010) and that flavonoids influence fatty acid biosynthesis via carbon source reallocation (Xuan et al., 2018). Photosynthesis and oxidative phosphorylation pathways might be related to fatty acid oxidation and the production of energy required for seed growth and development.

## Data availability statement

The raw BSR-seq, BSA-seq, and RNA-seq data were submitted to the NCBI Sequence Read Archive database (accession numbers: PRJNA668614, PRJNA834127, and PRJNA832681, respectively).

## Author contributions

ML, RW and JZ designed the experiments. ML, YZ, BL, YXS, JL, CZ, YW, HL, YMS, YF and LX performed the experiments and collected the data. ML, RW and JZ analyzed the data. ML wrote the manuscript. All authors contributed to the article and approved the submitted version.

## References

[B1] Alrefai R. Berke T. G. Rocheford T. R. (1995). Quantitative trait locus analysis of fatty acid concentrations in maize. Genome 38, 894–901. doi: 10.1139/g95-118 18470215

[B2] Blacklock B. J. Jaworski J. G. (2006). Substrate specificity of *Arabidopsis* 3-ketoacyl-CoA synthases. biochem. Biophys. Res. Commun. 346, 583–590. doi: 10.1016/j.bbrc.2006.05.162 16765910

[B3] Bolger A. M. Lohse M. Usadel B. (2014). Trimmomatic: a flexible read trimming tool for illumina sequence data. Bioinformatics 30, 2114–2120. doi: 10.1093/bioinformatics/btu170 24695404 PMC4103590

[B4] Chai Y. Hao X. Yang X. Allen W. B. Li J. Yan J.. (2012). Validation of *DGAT1-2* polymorphisms associated with oil content and development of functional markers for molecular breeding of high-oil maize. Mol. Breed. 29, 939–949. doi: 10.1007/s11032-011-9644-0

[B5] Chen X. Mao X. Huang J. Yang D. Wu J. Dong S.. (2011). KOBAS 2.0: a web server for annotation and identification of enriched pathways and diseases. Nucleic Acids Res. 39, 316–322. doi: 10.1093/nar/gkr483 PMC3125809 21715386

[B6] Dinges J. R. Colleoni C. Myers A. M. James M. G. (2001). Molecular structure of three mutations at the maize *sugary1* locus and their allele-specific phenotypic effects. Plant Physiol. 125, 1406–1418. doi: 10.1104/pp.125.3.1406 11244120 PMC65619

[B7] Dutta D. Harper A. Gangopadhyay G. (2022). Transcriptomic analysis of high oil-yielding cultivated white sesame and low oil-yielding wild black sesame seeds reveal differentially expressed genes for oil and seed coat colour. Nucleus 65, 151–164. doi: 10.1007/s13237-022-00389-0

[B8] Fang H. Fu X. Ge H. Zhang A. Shan T. Wang Y.. (2021). Genetic basis of maize kernel oil-related traits revealed by high-density SNP markers in a recombinant inbred line population. BMC Plant Biol. 21, 344. doi: 10.1186/s12870-021-03089-0 34289812 PMC8293480

[B9] Fang H. Fu X. Wang Y. Xu J. Feng H. Li W.. (2020). Genetic basis of kernel nutritional traits during maize domestication and improvement. Plant J. 101, 278–292. doi: 10.1111/tpj.14539 31529523

[B10] Flora L. F. Wiley R. C. (1972). Effect of various endosperm mutants on oil content and fatty acid composition of whole kernel corn *(Zea mays* l.). J. Amer Soc Hortic. Sci. 97, 604–607. doi: 10.21273/JASHS.97.5.604

[B11] Han Y. Xu G. Du H. Hu J. Liu Z. Li H.. (2017). Natural variations in stearoyl-acp desaturase genes affect the conversion of stearic to oleic acid in maize kernel. Theor. Appl. Genet. 130, 151–161. doi: 10.1007/s00122-016-2800-5 27717956

[B12] Hill W. G. (2005). A century of corn selection. Science 307, 683–684. doi: 10.1126/science.1105459 15692038

[B13] Hu Y. Colantonio V. Muller B. S. F. Leach K. A. Nanni A. Finegan C.. (2021). Genome assembly and population genomic analysis provide insights into the evolution of modern sweet corn. Nat. Commun. 12, 1227. doi: 10.1038/s41467-021-21380-4 33623026 PMC7902669

[B14] Jain B. P. Pandey S. (2018). WD40 repeat proteins: signalling scaffold with diverse functions. Protein J. 37, 391–406. doi: 10.1007/s10930-018-9785-7 30069656

[B15] Kim H. U. (2020). Lipid metabolism in plants. Plants 9, 871. doi: 10.3390/plants9070871 32660049 PMC7411677

[B16] Kim D. Langmead B. Salzberg S. L. (2015). HISAT: a fast spliced aligner with low memory requirements. Nat. Methods 12, 357–360. doi: 10.1038/nmeth.3317 25751142 PMC4655817

[B17] Kramer V. Shaw J. R. Senior M. L. Hannah L. C. (2015). The *sh2-R* allele of the maize *shrunken-2* locus was caused by a complex chromosomal rearrangement. Theor. Appl. Genet. 128, 445–452. doi: 10.1007/s00122-014-2443-3 25504539

[B18] Laurie C. C. Chasalow S. D. LeDeaux J. R. McCarroll R. Bush D. Hauge B.. (2004). The genetic architecture of response to long-term artificial selection for oil concentration in the maize kernel. Genetics 168, 2141–2155. doi: 10.1534/genetics.104.029686 15611182 PMC1448749

[B19] Li H. Durbin R. (2009). Fast and accurate short read alignment with burrows-wheeler transform. Bioinformatics 25, 1754–1760. doi: 10.1093/bioinformatics/btp324 19451168 PMC2705234

[B20] Li H. Peng Z. Yang X. Wang W. Fu J. Wang J.. (2013). Genome-wide association study dissects the genetic architecture of oil biosynthesis in maize kernels. Nat. Genet. 45, 43–50. doi: 10.1038/ng.2484 23242369

[B21] Liu M. Y. Burgos A. Ma L. Zhang Q. Tang D. Ruan J. (2017). Lipidomics analysis unravels the effect of nitrogen fertilization on lipid metabolism in tea plant *(Camellia sinensis* l.). BMC Plant Biol. 17, 165. doi: 10.1186/s12870-017-1111-6 29037151 PMC5644128

[B22] Liu N. Liu J. Fan S. Liu H. Zhou X. R. Hua W.. (2022). An integrated omics analysis reveals the gene expression profiles of maize, castor bean, and rapeseed for seed oil biosynthesis. BMC Plant Biol. 22, 153. doi: 10.1186/s12870-022-03495-y 35350998 PMC8966334

[B23] Liu H. Wei L. Zhu J. Zhang B. Gan Y. Zheng Y. (2022). Identification of *GmGPATs* and their effect on glycerolipid biosynthesis through seed-specific expression in soybean. Mol. Biol. Rep. 49, 9585–9592. doi: 10.1007/s11033-022-07852-w 36002658

[B24] Liu S. Yeh C. T. Tang H. M. Nettleton D. Schnable P. S. (2012). Gene mapping via bulked segregant RNA-seq (BSR-seq). PloS One 7, e36406. doi: 10.1371/journal.pone.0036406 22586469 PMC3346754

[B25] Love M. I. Huber W. Anders S. (2014). Moderated estimation of fold change and dispersion for RNA-seq data with DESeq2. Genome Biol. 15, 550. doi: 10.1186/s13059-014-0550-8 25516281 PMC4302049

[B26] Luo M. Lu B. Shi Y. Zhao Y. Wei Z. Zhang C.. (2022). A newly characterized allele of *ZmR1* increases anthocyanin content in whole maize plant and the regulation mechanism of different *ZmR1* alleles. Theor. Appl. Genet. 135, 3039–3055. doi: 10.1007/s00122-022-04166-0 35788748

[B27] Mckenna A. Hanna M. Banks E. Sivachenko A. Cibulskis K. Kernytsky A.. (2010). The genome analysis toolkit: a MapReduce framework for analyzing next-generation DNA sequencing data. Genome Res. 20, 1297–1303. doi: 10.1101/gr.107524.110 20644199 PMC2928508

[B28] Miklaszewska M. Zienkiewicz K. Inchana P. Zienkiewicz A. (2021). Lipid metabolism and accumulation in oilseed crops. OCL 28, 50. doi: 10.1051/ocl/2021039

[B29] Niu Y. F. Wang X. Hu D. X. Balamurugan S. Li D. W. Yang W. D.. (2016). Molecular characterization of a glycerol-3-phosphate acyltransferase reveals key features essential for triacylglycerol production in *Phaeodactylum tricornutum* . Biotechnol. Biofuels 9, 60. doi: 10.1186/s13068-016-0478-1 26973714 PMC4788866

[B30] Shen B. Allen W. B. Zheng P. Li C. Glassman K. Ranch J.. (2010). Expression of *ZmLEC1* and *ZmWRI1* increases seed oil production in maize. Plant Physiol. 153, 980–987. doi: 10.1104/pp.110.157537 20488892 PMC2899924

[B31] Tang B. Luo M. Zhang Y. Guo H. Li J. Song W.. (2021). Natural variations in the p-type ATPase heavy metal transporter gene *ZmHMA3* control cadmium accumulation in maize grains. J. Exp. Bot. 72, 6230–6246. doi: 10.1093/jxb/erab254 34235535

[B32] Tsuda K. Hake S. (2016). Homeobox transcription factors and the regulation of meristem development and maintenance. Plant Transcription Factors 14, 215–228. doi: 10.1016/B978-0-12-800854-6.00014-2

[B33] Vigeolas H. Waldeck P. Zank T. Geigenberger P. (2007). Increasing seed oil content in oil-seed rape *(Brassica napus* l.) by over-expression of a yeast glycerol-3-phosphate dehydrogenase under the control of a seed-specific promoter. Plant Biotechnol. J. 5, 431–441. doi: 10.1111/j.1467-7652.2007.00252.x 17430545

[B34] Vogt T. (2010). Phenylpropanoid biosynthesis. Mol. Plant 3, 2–20. doi: 10.1093/mp/ssp106 20035037

[B35] Wang K. Li M. Hakonarson H. (2010). ANNOVAR: functional annotation of genetic variants from high-throughput sequencing data. Nucleic Acids Res. 38, e164. doi: 10.1093/nar/gkq603 20601685 PMC2938201

[B36] Wang Z. H. Li S. X. Malhi S. (2008). Effects of fertilization and other agronomic measures on nutritional quality of crops. J. Sci. Food Agr 88, 7–23. doi: 10.1002/jsfa.3084

[B37] Wang F. Zheng T. Hu Z. Wu G. Lang C. Liu R. (2020). Overexpression of miR319a altered oil body morphogenesis and lipid content in *Arabidopsis* seeds. Plant Mol. Biol. Rep. 38, 531–537. doi: 10.1007/s11105-020-01217-y

[B38] Wu T. D. Reeder J. Lawrence M. Becker G. Brauer M. J. (2016). GMAP and GSNAP for genomic sequence alignment: enhancements to speed, accuracy, and functionality. methods mol. Biol 1418, 283–334. doi: 10.1007/978-1-4939-3578-9_15 27008021

[B39] Xiao Z. Tang F. Zhang L. Li S. Wang S. Huo Q.. (2021). The *Brassica napus* fatty acid exporter FAX1-1 contributes to biological yield, seed oil content, and oil quality. Biotechnol. Biofuels 14, 190. doi: 10.1186/s13068-021-02035-4 34587987 PMC8482660

[B40] Xuan L. Zhang C. Yan T. Wu D. Hussain N. Li Z.. (2018). TRANSPARENT TESTA 4-mediated flavonoids negatively affect embryonic fatty acid biosynthesis in *Arabidopsis* . Plant Cell Environ. 41, 2773–2790. doi: 10.1111/pce.13402 29981254

[B41] Yang Y. Benning C. (2018). Functions of triacylglycerols during plant development and stress. Curr. Opin. Biotechnol. 49, 191–198. doi: 10.1016/j.copbio.2017.09.003 28987914

[B42] Yang X. Guo Y. Yan J. Zhang J. Song T. Rocheford T.. (2010). Major and minor QTL and epistasis contribute to fatty acid compositions and oil concentration in high-oil maize. Theor. Appl. Genet. 120, 665–678. doi: 10.1007/s00122-009-1184-1 19856173

[B43] Yang R. Zhang L. Li P. Yu L. Mao J. Wang X.. (2018). A review of chemical composition and nutritional properties of minor vegetable oils in China. Trends Food Sci. Technol. 74, 26–32. doi: 10.1016/j.tifs.2018.01.013

[B44] Zafar S. Li Y. Li N. Zhu K. Tan X. (2019). Recent advances in enhancement of oil content in oilseed crops. J. Biotechnol. 301, 35–44. doi: 10.1016/j.jbiotec.2019.05.307 31158409

[B45] Zhang P. Allen W. B. Nagasawa N. Ching A. S. Heppard E. P. Li H.. (2012). A transposable element insertion within *ZmGE2* gene is associated with increase in embryo to endosperm ratio in maize. Theor. Appl. Genet. 125, 1463–1471. doi: 10.1007/s00122-012-1926-3 22772589

[B46] Zheng P. Allen W. B. Roesler K. Williams M. E. Zhang S. Li J.. (2008). A phenylalanine in DGAT is a key determinant of oil content and composition in maize. Nat. Genet. 40, 367–372. doi: 10.1038/ng.85 18278045

[B47] Zhu Z. Zhang Y. Wang J. Li X. Wang W. Huang Z. (2019). Characterization of sugar composition in Chinese royal jelly by ion chromatography with pulsed amperometric detection. J. Food Compos Anal. 78, 101–107. doi: 10.1016/j.jfca.2019.01.003

