# Supplementary material for: Genetic basis of the oil biosynthesis in ultra-high-oil maize grains with an oil content exceeding 20%
Source: Front Plant Sci. 2023 May 12;14:1168216. doi: 10.3389/fpls.2023.1168216 (PMC10213527; doi:10.3389/fpls.2023.1168216)
Supplement: Supplementary file 5 [file DataSheet_1.docx]

Supplementary Material

Genetic basis of the oil biosynthesis in ultra-high-oil maize grains with an oil content exceeding 20%

Meijie Luo^†^*, Baishan Lu^†^, Yaxing Shi^†^, Yanxin Zhao, Liu Junling, Chunyuan Zhang, Yuandong Wang, Hui Liu, Yamin Shi, Yanli Fan, Li Xu, Ronghuan Wang*, Jiuran Zhao*

† These authors contributed equally to this work.

Corresponding Author: [mjluo108@163.com](mailto:mjluo108@163.com); [maizezhao@126.com](mailto:maizezhao@126.com); [ronghuanwang@126.com](mailto:ronghuanwang@126.com)


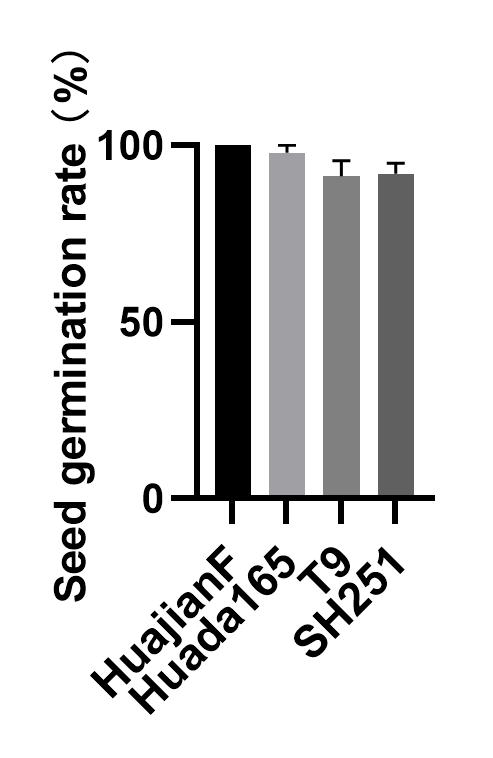


**Supplementary Figure 1.** Germination rates of HuajianF, Huada165, T9, and SH251 grains. The germination rate of 50 grains per maize line was calculated after a 7-day sand culture. Three biological replicates were analyzed. Data are presented as the mean ± SEM.


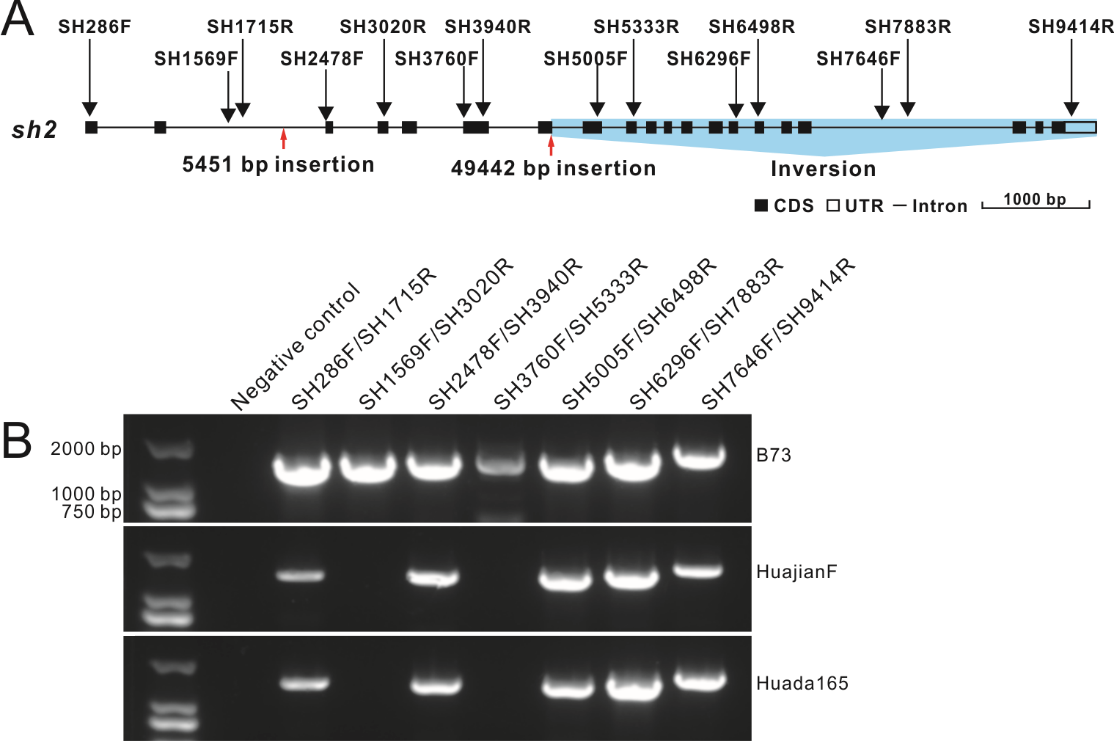


**Supplementary Figure 2** Analysis of the *Sh2* sequence in B73, HuajianF, and Huada165. (A) Locations of *Sh2* primer annealing sites and *sh2* mutation sites. (B) Comparison of the PCR products obtained for *Sh2* from B73, HuajianF, and Huada165.


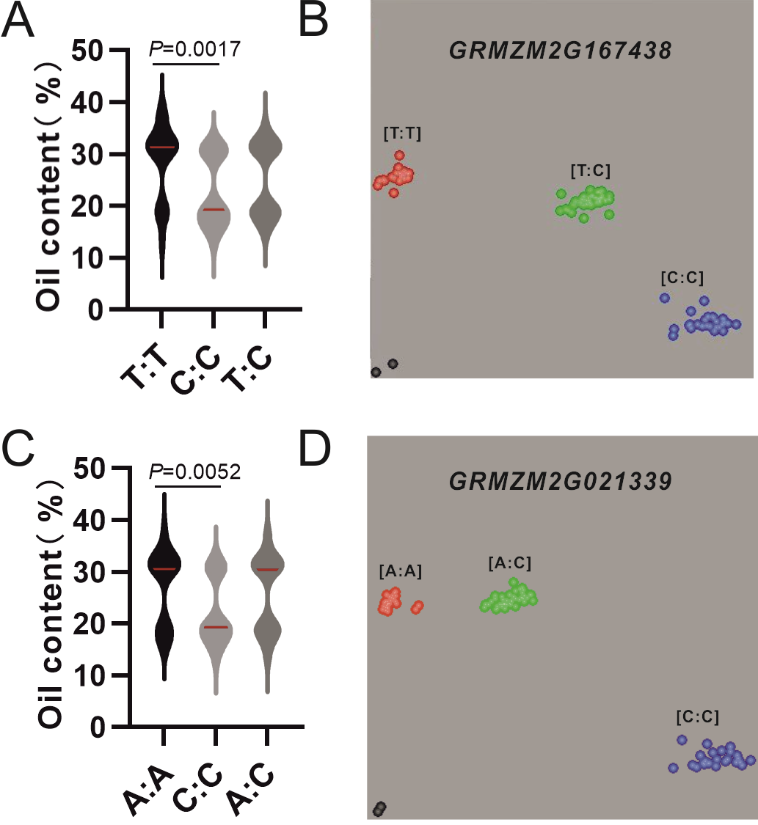


**Supplementary Figure 3.** KASP markers for *GRMZM2G167438* and *GRMZM2G021339* were significantly associated with the F_2_ maize grain (HuajianF × Huada165) oil content. (A and C) KASP assay genotyping results for 48 F_2_ plants (HuajianF × Huada165) with an extremely low oil content and 54 F_2_ plants (HuajianF × Huada165) with an extremely high oil content. (A) KASP marker genotyping results for *GRMZM2G167438*. (C) KASP marker genotyping results for *GRMZM2G021339*. (B and D) Association between the genotyping results and the grain oil contents.

**Supplementary Table 1** Information regarding the 189 sweet maize inbred lines used for genotyping (See supplementary Table 1 excel file)

**Supplementary Table 2** Primers used in this study (See supplementary Table 2 excel file)

**Supplementary Table 3** Maize grain oil content-related QTLs identified by BSA-seq (See supplementary Table 3 excel file)

**Supplementary Table 4** Differentially expressed genes identified in this study (See supplementary Table 4 excel file)
